# Hepatoprotective effects of peach gum polysaccharides against alcoholic liver injury: moderation of oxidative stress and promotion of lipid metabolism

**DOI:** 10.3389/fnut.2023.1325450

**Published:** 2024-01-11

**Authors:** Bingjie Zhou, Pinpin Liu, Xiangao Yao, Huijie Cao, Hang Zhu, Qiao Wang, Yan Liu, Min Fang, Yongning Wu, Zhiyong Gong

**Affiliations:** ^1^Hubei Key Laboratory for Processing and Transformation of Agricultural Products, Key Laboratory for Deep Processing of Major Grain and Oil (The Chinese Ministry of Education), Food Safety Research Center for Key Research Institute of Humanities and Social Sciences of Hubei Province, Wuhan Polytechnic University, Wuhan, China; ^2^Suizhou Center for Disease Control and Prevention, Hubei Province, China; ^3^NHC Key Laboratory of Food Safety Risk Assessment, China National Center for Food Safety Risk Assessment, Food Safety Research Unit (2019RU014) of Chinese Academy of Medical Sciences, Beijing, China

**Keywords:** peach gum polysaccharides, structural analysis, antioxidant activity, alcoholic liver damage, metabolomic

## Abstract

Natural polysaccharides extracted from plants have received increasing attention due to their rich bioactivity. In our study, peach gum polysaccharides (PGPs) were extracted by water extraction-alcohol precipitation method. PGPs are typical pyranose polysaccharides with a mean molecular weight of 3.68 × 10^6^ g/mol. The antioxidant activity and hepatoprotective capacity of PGPs were studied. *In vitro*, assays showed that PGPs scavenged DPPH, OH, and O_2_^–^ in a dose-dependent manner. PGPs exhibited antioxidative properties against alcohol-induced HL7702 cells, as evidenced by the normalization of MDA, SOD, ROS, and GSH levels. To further elucidate the hepatoprotective mechanism of PGPs, we carried out *in vivo* experiments in male mice. PGPs exerted hepatoprotective effects in alcohol liver disease (ALD) mice by exerting antioxidant effects, decreasing the inflammatory response and modulating lipid metabolism. In addition, metabolomic analysis indicated that PGPs mainly regulate D-glutamine and D-glutamate metabolism, alanine, aspartate and glutamate metabolism, and arginine biosynthesis to promote hepatic metabolism and maintain body functions. Overall, this study revealed that the hepatoprotective mechanism of PGPs against ALD might be associated with the regulation of oxidative stress and lipid metabolism.

## 1 Introduction

Peach gum (PG), a gelatinous substance secreted by the bark of the peach tree, is recorded in the *Compendium of Materia Medica* as alleviating pain (1). Generally, the key component of PG is a macromolecule polysaccharide (2). PGPs consist of (1→3) and (1→6) linked Galp unit backbones and highly branched macromolecular structures (3). PGPs with highly branched macromolecular structures show favorable bioactivity, emulsifying performance, and antioxidant and antibacterial activity (1). Modern scientific research has found that PGPs exert hypoglycemic and hypolipidemic properties, as well as improve intestinal flora and enhance human immunity (4).

Alcoholic liver disease (ALD) is a clinical form of liver injury due to excessive alcohol intake. The latest epidemiological survey found that ALD is still one of the most common diseases that seriously endangers public health worldwide, showing an increasing yearly trend (5). Alcohol intake increases the amount of nicotinamide adenine dinucleotide/NAD+ produced by the oxidation of ethanol to acetaldehyde in hepatocytes, thereby disrupting fatty acid oxidation. The natural course of alcoholic liver disease follows the pattern of steatosis - alcoholic hepatitis - fibrosis - cirrhosis - hepatocellular carcinoma. Hepatic steatosis, defined histologically as intracellular fat deposition, is an early reversible stage of ALD due to ethanol exposure (6–8). The liver, the main site of ethanol metabolism, contains high levels of alcohol dehydrogenase (ADH), which catalyzes the oxidation of ethanol to the toxic metabolite (9). Oxidative metabolites produced by alcohol metabolism, including acetaldehyde and free radicals, dominate the pathological progression of the alcoholic liver (10). The large morbidity and mortality rates associated with ALD are primarily prevented through political measures to reduce the availability of alcohol (7). However, a limited number of drugs with low adverse effects are available to mitigate alcoholic liver injury (5). Therefore, current research is focused on finding and utilizing natural active ingredients with low toxicity and high efficiency.

Polysaccharides derived from some organisms exhibit excellent anti-ALD activity (5, 11). The hepatic metabolic burden can be attenuated due to the antioxidative and hypolipidemic properties of polysaccharides. Furthermore, the anti-ALD characteristics of polysaccharides were attributed to hepatoprotective effects in regulating fatty acid metabolism and reducing reactive oxygen species production (12). Nepali et al. reported that polysaccharides inhibited the mRNA expression of CYP2E1 and NADPH oxidase in ethanol-fed mice, which was considered to moderate oxidative stress and lipid accumulation induced by alcohol intake (13). Unlike drugs, natural PGPs can be developed as functional foods for daily intake to provide preventive control of ALD. Additionally, the biological activity and high yield of PGPs that have been characterized make them broad prospects for development. However, the alleviation of ALD by PGPs has never been reported. The great exploitation potential of PGPs provides more profitable possibilities for farmers. Therefore, we focused on the protective mechanism of ALD to investigate the bioactivity of PGPs.

**FIGURE 1:**
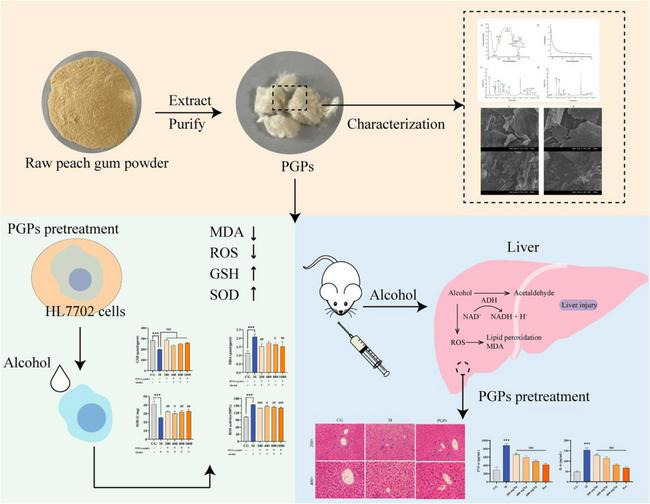
Preliminarily explored the protective mechanism of PGPs against ALD.

In our study, PGPs were extracted with hot water, and their monosaccharide composition and molecular weight distribution were characterized. The hepatoprotective activity of PGPs on ALD models was evaluated *in vitro* and *in vivo*. Metabolomics techniques were used to explore potential mechanisms of action. Our results suggest that PGPs exert protective effects against ALD in terms of decreasing oxidative stress and regulating lipid metabolism. We preliminarily explored the protective mechanism of PGPs against ALD, aiming to provide a theoretical basis for the development of related functional products as in Figure 1.

## 2 Materials and methods

### 2.1 Materials and chemicals

Raw peach gum powder was provided by Suizhou Peach Gum Industry, Hubei, China. Methanol, acetonitrile, and formic acid were chromatographically pure from Merck Chemical Co. (Darmstadt, Germany). Trichloromethane, anhydrous ethanol, sulfuric acid and standard monosaccharides were purchased from Sinopharm Chemical Reagents Co., Ltd. (Shanghai, China). The chemicals and reagents used in our experiment were analytically pure.

Malonaldehyde (MDA), glutathione (GSH), superoxide dismutase (SOD), catalase (CAT), γ-gamma glutamyl transpeptidase (γ-GGT), and triglyceride (TG) assay kits were purchased from Nanjing Jiancheng Biological Engineering Institute. Aspartate aminotransferase (AST), alanine aminotransferase (ALT), alcohol dehydrogenase (ADH), and acetaldehyde dehydrogenase (ALDH) assay kits were purchased from Solarbio Science and Technology Co. Reactive oxygen species (ROS) assay kits and ELISA kits were purchased from Beyotime Biotechnology Institute.

### 2.2 Preparation of PGPs

After passing through a100 mesh sieve, the raw peach gum powder was added to Milli-Q water at 1:100 (g/mL), stirred at 90 °C for 2 h, extracted twice, and centrifuged at 4500 rpm for 15 min. The supernatant was concentrated to 1/5 using a rotary evaporator, and four times the volume of anhydrous ethanol was added. The peach gum-ethanol solution was placed at 4 °C overnight to precipitate the crude polysaccharides. The crude polysaccharide was redissolved in Milli-Q water at 1:25 (g/mL) and deproteinized by Sevage reagent (chloroform: n-butyl alcohol = 4:1, V/V). Dialysis was performed using a dialysis bag with a molecular weight cutoff of 14000 Da for 48 h. Vacuum freeze-drying was used to obtain PGPs.

### 2.3 Characterization of PGPs

Carbohydrates in PGPs were determined by the phenol-sulfate method using glucose as a standard. The structure of PGPs was observed by scanning electron microscope (SEM). A total of 1 μg/mL PGPs-water solution was dropped on a clean and flat mica substrate, naturally air-dried and observed using an atomic force microscope equipped with a silicon SPM probe (AFM; Nanosurf, CH) (14).

The molecular weight (M_*w*_) of PGPs was estimated by high-performance size-exclusion chromatography (HPSEC) (11). High-performance anion-exchange chromatography (HPAEC) equipped with a pulsed amperometric detector (PAD; Dionex ICS 5000 + system, USA) was used to determine the monosaccharide composition according to the method of Wang et al. (15).

The PGPs (2 mg) were fully ground with 200 mg of KBr under infrared light irradiation drying conditions and scanned in the wavelength range of 400–4000 cm^–1^ using a Fourier transform infrared spectrometer (FTIR; PerkinElmer, USA). An ultraviolet-visible spectrophotometer (UV-Vis; Thermo, USA) was used to scan at 190–400 nm.

### 2.4 *In vitro* free radical scavenging assay

#### 2.4.1 2-Diphenyl-picryl hydrazine free radical (DPPH) scavenging ability

The 2-diphenyl-picryl hydrazine free radical (DPPH) scavenging ability was determined according to the method of Yao et al. (16) with slight modifications. PGPs solutions ranging from 1 to 10 mg/mL were prepared. Then, 0.2 mL DPPH radical solution (0.4 mol/L in anhydrous ethanol) and 2 mL deionized water were successively added to 1.0 mL PGPs solution. The solution was mixed and left in the dark for 30 min at room temperature. The control (deionized water instead of PGPs solutions) was used as a calibrator, and the absorbance was measured at 517 nm wavelength. Vitamin C was used as a positive control.


(1) $$\mathrm{DPPH}\,\mathrm{scavenging}\,\mathrm{ability}=\left[1-\frac{\left({\text{A}}_{1}-{\text{A}}_{2}\right)}{{\text{A}}_{0}}\right]\times 100\%$$

where A_0_ represents the blank absorbance (water instead of PGPs solution), A_1_ represents the absorbance of the complete reflection system, and A_2_ represents the absorbance of the reaction system without DPPH solution (using water instead).

#### 2.4.2 Hydroxyl radical (OH) scavenging ability

Hydroxyl radical (OH) scavenging ability was determined according to the method of Long et al. (17) with slight modifications. Two milliliters of FeSO_4_ solution (6 mmol/L), 2 mL of H_2_O_2_ solution (6 mmol/L) and 2 mL of salicylic acid solution (6 mmol/L) were successively added to 2 mL of PGPs solution (1–10 mg/mL). After standing for 30 min, the absorbance was measured at a wavelength of 510 nm. Vitamin C was used as a positive control.


(2) $$\mathrm{OH}\,\mathrm{scavenging}\,\mathrm{ability}=\left[1-\frac{\left({\text{A}}_{1}-{\text{A}}_{2}\right)}{{\text{A}}_{0}}\right]\times 100\%$$

where A_0_ represents the blank absorbance (water instead of PGPs solution), A_1_ represents the absorbance of the complete reflection system, and A_2_ represents the absorbance of the reaction system without H_2_O_2_ solution (using water instead).

#### 2.4.3 Superoxide anion free radical (O_2_^–^) scavenging ability

Referring to the method of Zhang et al. (18), the O_2_^–^ scavenging ability was determined. Then, 4.5 mL of Tris-HCl buffer solution (50 mmol/L, pH 8.2) and 0.4 mL of pyrogallol solution (25 mmol/L) were successively added to 2 mL of PGPs solution (0.1–1.0 mg/mL). After a 25-min water bath at room temperature, 1 mL of HCl (8 mmol/L) solution was added, and the absorbance was quickly measured at 325 nm. Vitamin C was used as a positive control.


(3) $${\text{O}}_{2}^{-}\,\mathrm{scavenging}\,\mathrm{ability}=\left[1-\frac{\left({\text{A}}_{1}-{\text{A}}_{2}\right)}{{\text{A}}_{0}}\right]\times 100\%$$

where A_0_ represents the blank absorbance (water instead of PGPs solution), A_1_ represents the absorbance of the complete reflection system, and A_2_ represents the absorbance of the reaction system without pyrogallol solution (using water instead).

### 2.5 *In vitro* cell experiments

#### 2.5.1 Cell culture and treatment

HL7702 human hepatocytes were obtained from the China Center for Type Culture Collection. HL7702 cells were cultured in RPMI 1640 medium supplemented with 10% fetal bovine serum (FBS) and 1% penicillin-streptomycin solution in sterile culture flasks. Cells were maintained at 37 °C with 5% CO_2_ in a CO_2_ incubator (Memmert, Germany).

#### 2.5.2 Cell viability assay

The methyl thiazolyl tetrazolium (MTT) method was used to individually determine cell viability after PGPs and alcohol intervention. Then, 0 μg/mL, 12.5 μg/mL, 25 μg/mL, 50 μg/mL, 100 μg/mL, 200 μg/mL, 400 μg/mL, 800 μg/mL, and 1000 μg/mL PGP solutions and 0, 0.5, 1, 2, 3, and 4% alcohol were added to RPMI 1640 medium, respectively. Cells were seeded into 96-well plates and cultured for 24 h to confirm cell apposition. The old medium was discarded and the cells were washed with PBS before the addition of configured PGPs solution or alcohol. After incubation in PGPs solution for 24 h or in ethanol for 12 h, 10 μL/well of MTT solution was added to the plate for 4 h of incubation. Absorbance values were measured at 570 nm after full dissolution with 100 μL/well of methyl saliva.

#### 2.5.3 Detection of intracellular MDA, GSH, SOD, and ROS

According to the MTT experiment results, 200 μg/mL, 400 μg/mL, 800 μg/mL, and 1000 μg/mL were selected as the PGPs solution concentrations for the experimental group, and 3% alcohol was used for modeling. Cells were seeded into 6-well plate, treated with PGPs solution for 24 h, removed and washed with PBS, followed by replacement with 3% alcohol for 12 h. Cells were collected. The intracellular MDA, GSH, SOD, and ROS contents were determined according to the manufacturer’s protocol. The control, model, and PGPs pretreatment groups were named CG, M, 200, 400, 800, and 1000, respectively.

### 2.6 Hepatoprotective effects of PGPs *in vivo*

#### 2.6.1 Animal treatment and modeling

**FIGURE 2:**
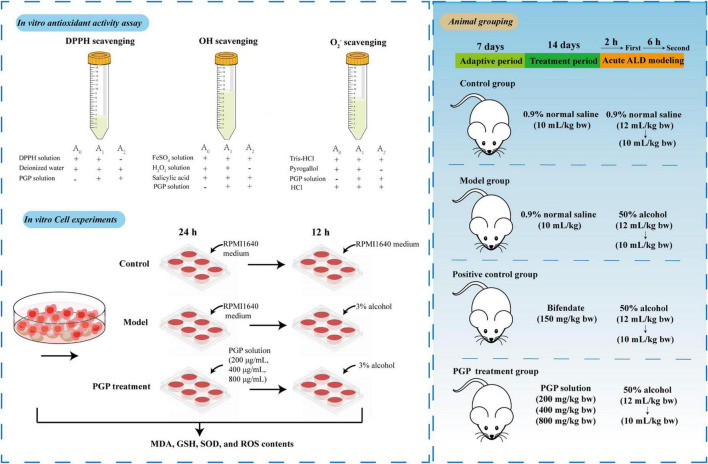
Scheme for the study of the protective mechanism of PGPs against alcoholic liver injury.

Acute alcohol exposure after polysaccharide intervention is a widely used model of ALD (19–21). Therefore, we selected this type of acute ALD model for experiments to better simulate the body’s resistance to ALD after daily intake of PGPs. Forty-eight SPF KM male mice (No. 42000600048880) were obtained from Hubei Laboratory Animal Research Center. Mice were maintained in a specific pathogen-free environment with room temperature at 25 ± 4°C, 60 ∼ 70% relative humidity, and 12 h light alternated. After 1 week of adaptive feeding, the mice were randomly divided into 6 groups, including the control group (CG), model group (M), 150 mg/kg bifendate positive control group (Pos), 200 mg/kg PGPs group (200), 400 mg/kg PGPs group (400), and 800 mg/kg PGPs group (800), with 8 mice in each group. Gavage was administered daily at 10 am for 14 consecutive days at the above dose, and mice in the CG and M groups were treated with 0.9% saline instead of PGPs or bifendate. Two hours after the last gavage, mice in the model and PGP groups were administered 50% ethanol (12 mL/kg body weight), and 6 h later, 50% ethanol was gavaged again at a dose of 10 mL/kg (22). Equal amounts of 0.9% normal saline were given to the control group. Experimental mice were sacrificed after overnight fasting, and blood and liver were collected. The experimental design and operating procedures were approved by the Animal Welfare Ethics Committee of Wuhan Polytechnic University (WPU202209001). The experimental plan was shown in Figure 2.

#### 2.6.2 Biochemical assays

Precooled saline was added to the liver tissue at a ratio of 9:1, and the homogenate was prepared using a high-throughput tissue grinder. Blood was centrifuged at 3000 rpm for 10 min at 4 °C and the supernatant serum was collected.

The contents of ALT, AST, and γ-GGT were measured in the serum. The oxidative stress indicators GSH, MDA, CAT, and SOD and the alcohol-metabolizing enzyme (ADH, ALDH) and TG were measured in liver homogenates using analytical kits. The levels of lipopolysaccharide (LPS), tumor necrosis factor-α (TNF-α), and interleukin-6 (IL-6) in liver homogenate were measured by ELISA.

#### 2.6.3 Histopathological observation

Liver tissue of 5 mm × 5 mm × 3 mm size was harvested from the middle-left lobe of the mouse liver and fixed in tissue fixative solution (4% paraformaldehyde) for hematoxylin-eosin (H&E) staining and oil red staining.

#### 2.6.4 Untargeted metabolomics analysis

A mixture of organic solvent (acetonitrile: methanol: water = 2:2:1, v/v/v) was added to 50 mg of liver tissue in a 2 mL EP tube and fully ground for 5 min using a high-throughput tissue grinder. Tissue homogenates were vortexed for 10 min followed by 30 min of sonication in an ice water bath. After centrifugation at 12,000 rpm for 15 min at 4 °C, the supernatant was aspirated, and nitrogen was blown to dryness using an automatic nitrogen blower at room temperature. The dried sample was redissolved in 200 μL of acetonitrile-water (1:1, v/v), centrifuged at 15,000 rpm for 10 min at 4 °C to obtain the supernatant, and transferred to an injection bottle equipped with a lined tube for measurement. QC samples, consisting of all liver samples mixed in equal volumes, were used to test the stability of the analytical method.

Untargeted metabolomics analysis was performed on an Orbitrap Exploris 120 mass spectrometer coupled with a Vanquish UHPLC system (Thermo Fisher Scientific). Metabolites were separated using a Waters ACQUITY HSS T3 column at a flow rate of 0.4 mL/min. The column temperature was 45 °C, and the injection volume was 8 μL. Mobile phase A was 0.1% formic acid water, and mobile phase B was acetonitrile. The gradient elution program was as follows: 5% B for 0∼1 min, 5–30% B for 1∼3 min, 30–80% B for 3∼5 min, 80–100% B for 5∼13 min, 100–5% B for 13∼13.1 min, and 5% B for 15 min. Mass spectra were obtained in full scan-ddms2 mode with an H-ESI ion source. Mass spectrometry conditions were as follows: spray voltage, 4000 V in the positive ion mode and 3500 V in the negative ion mode; sheath gas: 30 Arb; ion transfer tube temp: 320 °C; vaporizer temp: 350 °C; orbitrap resolution: 12000. For sequence acquisition, a QC sample was interspersed every six needles.

### 2.7 Statistical analysis

Data analysis was conducted by using Microsoft Office Excel 2016 (Microsoft., USA) and SPSS Statistics 27 (Microsoft., USA). MS-DIAL 4.7 was used to perform peak alignment, retention time correction, and peak area extraction, parameters were shown in Supplementary Table 1. SIMCA-P 14.1 and R 4.2.3 software were used for multivariate statistical analysis. Metabolic pathway analysis was performed in Metaboanalyst 5.0.

## 3 Results

### 3.1 Molecular weight and monosaccharide composition of PGPs

**FIGURE 3:**
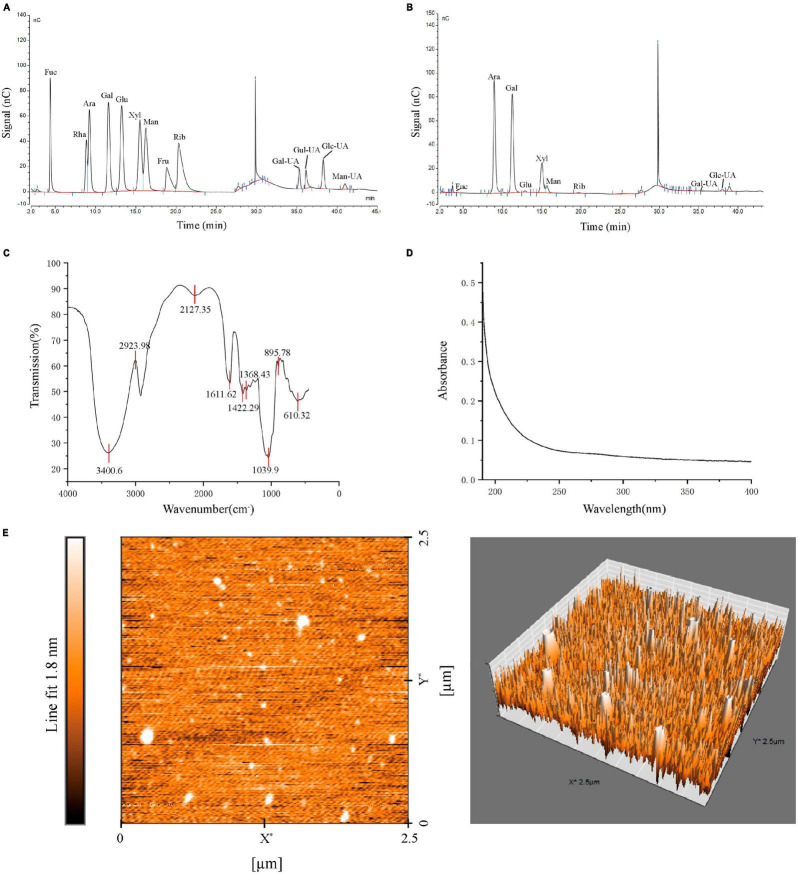
Characterization of PGPs. **(A)** Monosaccharide composition of standard. **(B)** Monosaccharide composition of PGPs. **(C)** FT-IR spectra. **(D)** UV-Vis spectra. **(E)** AFM images of PGPs.

In our study, PGPs contained 92.12 ± 0.85% total carbohydrate. HPSEC analysis showed that PGPs contained three fractions, with molecular weights estimated to be 5.010 × 10^6^ g/mol (68.5%), 7.888 × 10^5^ g/mol (28.3%), and 7.098 × 10^5^ g/mol (3.2%) (Supplementary Figure 2). The mean molecular weight of PGPs was 3.68 × 10^6^. The monosaccharide composition of the PGPs is shown in Table 1, consisting mainly of Ara, Gal, Xyl, Glc-UA, and Man (Figures 3A,B).

**TABLE 1:** Monosaccharide contents of PGPs.

Monosaccharide composition	Content (μ g/mg)	Percentage (%)
Arabinose (Ara)	208.93	43.90
Galactose (Gal)	167.60	35.12
Xylose (Xyl)	53.98	11.47
Glucuronic acid (Glc-UA)	17.52	3.72
Mannose (Man)	16.41	3.49
Glucose (Glu)	2.29	0.49
Galacturonic acid (Gal-UA)	1.94	0.41
Ribose (Rib)	1.81	0.38
Fucose (Fuc)	0.21	0.04

### 3.2 Spectral analysis and morphology of PGPs

The FT-IR spectrum of PGPs is shown in Figure 3C. The characteristic peak at 3400.6 cm^–1^ was assigned to the O-H stretching vibration of saccharide. The weak absorption peaks at 2924.0 cm^–1^ attributed to the tensile vibration of C-H (CH, CH2, CH3) are typical of polysaccharides. In addition, the weak vibrations at 1611.6 cm^–1^ and 1422.3 cm^–1^ were attributed to C = O(-COOH) symmetric stretching vibrations. The characteristic peak at 1039.9 cm^–1^ indicated the presence of pyranoside in PGPs (23). The characteristic absorption peak at approximately 895 cm^–1^ indicated that the glycosyl residues of PGPs are mainly β-type glycosidic bonds (24). In conclusion, PGPs are typical pyranose polysaccharides linked by a β-glycosidic bond. Figure 3D shows the UV-vis spectrum of PGPs at 190–400 nm, without obvious absorption peaks appearing at 200–280 nm, indicating the absence of nucleic acids and proteins in PGPs (4).

Peach gum polysaccharides are lamellar structures with a relatively smooth surface containing pore-like structures resulting from water volatilization (Supplementary Figure 1). AFM images showed that the chain morphology of PGPs was irregular spherical conformations with the height about 1.0 nm (Figure 3E). This result is consistent with previously study that reported on the structure of PGPs (25). Due to van der Waals forces between polysaccharide chains or hydrogen bond interactions between molecules, polysaccharide chains are wound to form a spherical structure (14).

### 3.3 Scavenging ability for DPPH, OH, and O_2_^–^

**FIGURE 4:**
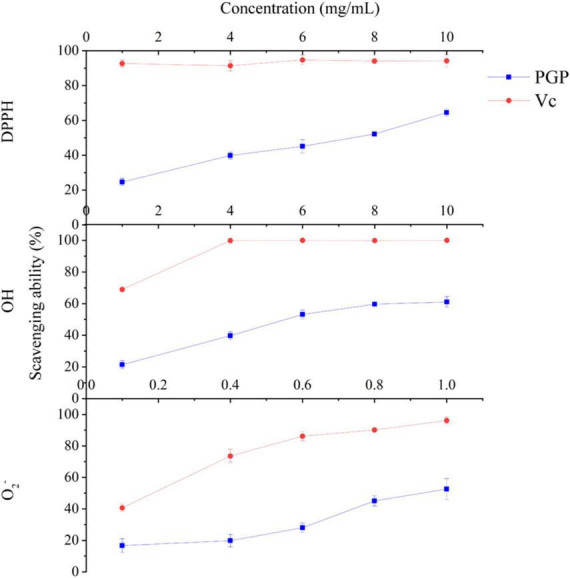
*In vitro* antioxidant capacity of PGPs.

The DPPH, OH and O_2_^–^ scavenging abilities of PGPs are shown in Figure 4, and excellent antioxidant ability was observed. The ability of PGPs to scavenge free radicals was dose-dependently enhanced. As the concentration of PGPs gradually increased from 1 mg/mL to 10 mg/mL, the DPPH scavenging rate increased from 24.59 ± 2.17% to 64.48 ± 2.06%, and the OH scavenging rate increased from 21.44 ± 2.52% to 61.11 ± 3.39%. For O_2_^–^, the free radical scavenging ability of PGPs increased from 16.77 ± 4.39% to 52.56 ± 6.73% at concentrations ranging from 0.1 mg/mL to 1.0 mg/mL. The IC_50_ values of scavenging ability for DPPH, OH, and O_2_^–^ were 6.93 mg/mL, 6.45 mg/mL, and 0.99 mg/mL, respectively. This result confirms the antioxidant ability of PGPs *in vitro*.

### 3.4 Effects of PGPs and alcohol on the viability of HL7702 cells

**FIGURE 5:**
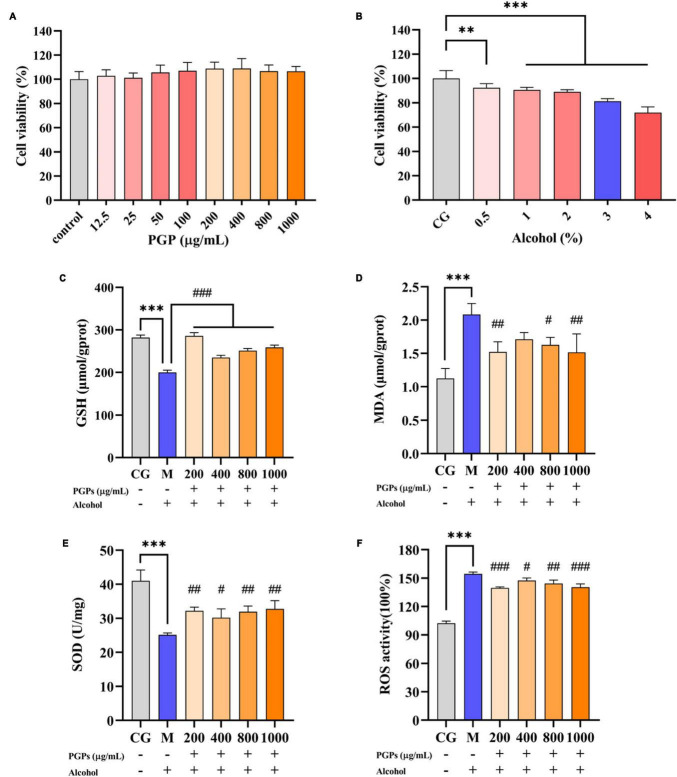
Protective effect of PGPs on alcohol-induced HL7702 cells. **(A)** Viability of HL7702 cells cultured with PGPs for 24 h. **(B)** Viability of HL7702 cells cultured with different concentrations of alcohol for 12 h. **(C)** GSH contents. **(D)** MDA contents. **(E)** SOD contents. **(F)** ROS contents. ** and *** indicate *P* < 0.01 and *P* < 0.001, respectively, for Group M compared with group CG. #, ##, and ### indicate *P* < 0.05, *P* < 0.01, and *P < 0.001*, respectively, for group PGPs compared with group M.

As shown in Figure 5A, there was no significant difference in the viability of HL7702 cells exposed to different concentrations of PGPs (12.5, 25, 50, 100, 200, 400, 800, and 1000 μg/mL), indicating that the interference of PGPs on cell activity was limited. The cytotoxic effects induced by alcohol on cell viability were evaluated (Figure 5B) to select the intervening concentration of alcohol in the cell assay. The percentage of cell vitality decreased from 92.33% to 71.88% when the alcohol content was steadily increased from 0.5% to 4%, and a significant (*P* < 0.01) dose-dependent decrease was observed. The cell viability was 81.25% at 3% alcohol concentration, which could effectively establish an acute ALD cell model without causing a large number of cell death. Therefore, a 3% alcohol concentration and incubation for 12 h was selected as the *in vitro* alcohol damage to HL7702 cells to guarantee the quantity of cells for further tests.

### 3.5 Effect of PGPs on oxidative stress in HL7702 cells

HL7702 cells were cultured with 3% alcohol medium for 12 h. Compared with the CG group, a significant decrease (*P* < 0.001) of 29.09% and 56.57% in the intracellular GSH and SOD contents was observed in the M group (Figures 5C, E). The MDA and ROS contents in M group were significantly increased (*P* < 0.001) by 84.41% and 51.06%, respectively (Figures 5D, F). Cells showed significant oxidative damage in the presence of 3% alcohol. Under the protective influence of PGPs, the contents of GSH (*P* < 0.001) and SOD (*P* < 0.05) increased significantly, and the contents of MDA (*P* < 0.05) and ROS (*P* < 0.05) decreased significantly, demonstrating a considerable antioxidative stress effect.

### 3.6 Hepatoprotective effect of PGPs in mice

**FIGURE 6:**
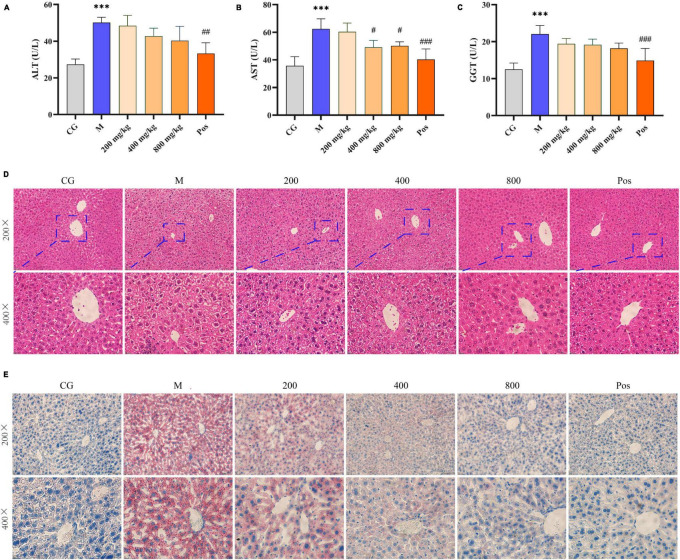
Serum levels of AST **(A)**, ALT **(B)**, and γ-GGT **(C)**. Morphological observation of the liver in alcohol-induced mice. **(D)** H&E staining. **(E)** Oil red staining. *** indicate *P* < 0.001, respectively, for Group M compared with group CG. #, ##, and ### indicate *P* < 0.05, *P* < 0.01, and *P < 0.001*, respectively, for group PGPs compared with group M.

Serum levels of ALT, AST, and γ-GGT were used to assess liver function (Figures 6A–C). The contents of ALT, AST, and γ-GGT in M group were significantly increased under alcohol intervention (*P* < 0.001), indicating liver injury in the mice. Although the effects of PGPs on ALT and γ-GGT levels were not significant, PGPs exhibited a dose-dependent reduction in alcohol-induced dysregulation of ALT, AST, and γ-GGT levels. The AST levels in the serum of alcohol-induced mice were significantly reduced under the effect of 400 and 800 mg/kg PGPs (*P* < 0.05).

The HE-stained sections of liver tissue from each group are shown in Figure 6D. In the CG group, the hepatocytes had normal structure and regular morphology, and were neatly arranged around the central vein, with regular round nuclei and visible cell gaps. In the M group, the hepatocytes were disordered, swollen or even disintegrated, the boundary was fused, and the hepatocyte blisters degenerated into honeycomb-like structures. Hepatocyte damage was significantly ameliorated in the PGPs and Pos groups, and the degree of hepatocyte swelling, disintegration, and vacuolar degeneration was reduced. The PGPs-800 group showed less damage than the low-dose group, and the hepatocytes were relatively neatly arranged with clear cell gaps.

**FIGURE 7:**
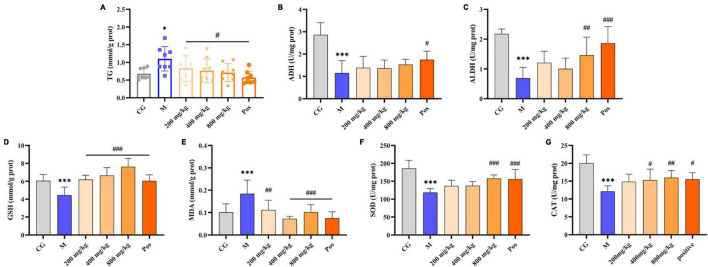
Effects of PGPs on biochemical constituents in mouse livers. **(A)** TG contents. **(B)** ADH contents. **(C)** ALDH contents. **(D)** GSH contents. **(E)** MDA contents. **(F)** SOD contents. **(G)** CAT contents. * and *** indicate *P* < 0.05 and *P* < 0.001, respectively, for Group M compared with group CG. #, ##, and ### indicate *P* < 0.05, *P* < 0.01, and *P < 0.001*, respectively, for group PGPs compared with group M.

The oil red-stained sections of liver tissue from each group are shown in Figure 6E. No obvious red lipid droplets were observed in the CG group, while dense and extensive red lipid droplets were observed in the livers of M group, indicating that lipid metabolism disorder occurred in the ALD mice. In contrast, the accumulation of lipid droplets in the livers of PGPs-pretreated mice was mitigated and the degree of denseness was also reduced. Liver TG levels were examined to further confirm the protective effect of PGPs against alcohol-induced disorders of hepatic lipid metabolism (Figure 7A). The TG contents were significantly decreased (*P* < 0.05) in the PGPs and Pos groups, with reductions of 35.53% and 48.03% in the PGPs-800 and Pos groups, respectively. PGPs decreased TG contents in a dose-dependent manner, and TG content in PGPs-800 group was 104.30% of that in CG group. TG content of Pos group decreased to 84.13% of CG group. From histopathologic observations, the number of red lipid droplets in the PGPs-800 group approached that of the CG group, while the Pos group contained fewer lipid droplets than the CG group. The drug bifendate (Pos), which is directed at reducing aminotransferases, increases hepatocyte activity and accelerates lipid droplet metabolism.

### 3.7 Alcohol metabolizing enzymes, oxidative stress markers and inflammatory factors in mouse livers

**FIGURE 8:**
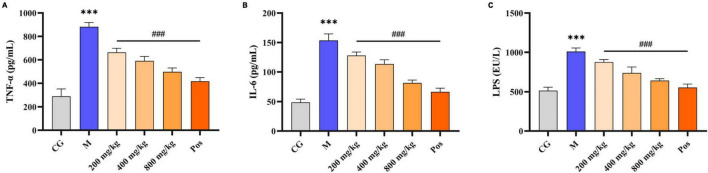
Effects of PGPs on biochemical constituents in mouse livers. **(A)** TNF-α contents. **(B)** IL-6 contents. **(C)** LPS contents. *** indicate *P* < 0.001, for Group M compared with group CG. ### indicate *P < 0.001*, for group PGPs compared with group M.

Under alcohol intervention, significant differences (*P* < 0.001) in alcohol metabolizing enzymes (ADH, ALDH), oxidative stress indices (MDA, GSH, SOD, CAT), and inflammatory factors (TNF-α, IL-6, LPS) were observed in M group compared with CG group (Figures 7, 8). Pretreatment with PGPs and bifendate significantly attenuated (*P* < 0.01) alcohol-induced abnormal increases in MDA, TNF-α, IL-6, and LPS and abnormal decreases in GSH, SOD, and CAT. A dose-dependent trend was observed in the changes in TNF-α, IL-6, LPS, GSH, SOD, and CAT contents. Compared with M group, the contents of MDA, TNF-α, IL-6 and LPS in PGPs-800 group were decreased by 44.67%, 43.64%, 26.16% and 36.70%, respectively. The contents of GSH, SOD, and CAT in the PGPs-800 group were increased by 71.73%, 33.21%, and 34.49%, respectively. The ADH and ALDH contents were significantly decreased by 56.22% and 68.3% in M group compared to CG group (*P* < 0.001). In contrast, the intervention of 200 mg/kg and 400 mg/kg PGPs in improving ethanol metabolism’s enzymatic activity were insignificant (*P* > 0.05). ALDH content was significantly (*P* < 0.01) increased by the intervention of 800 mg/kg of PGPs.

### 3.8 Metabolomics was used to verify the hepatoprotective effect of PGPs

**FIGURE 9:**
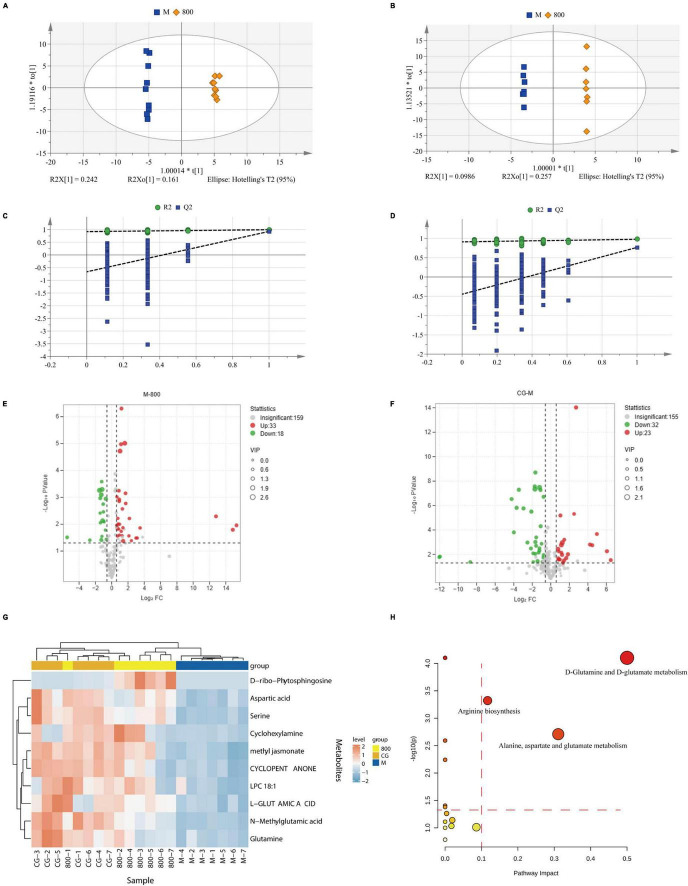
Multivariate statistical analysis and pathway analysis of metabolomics. OPLS-DA scores plot of M vs. 800 in positive **(A)** and negative **(B)**. Permutation tests of M vs. 800 in positive **(C)** and negative **(D)**. Volcano plot of M vs. 800 **(E)** and CG vs. M **(F)**. **(G)** Heatmap of differential metabolites. **(H)** Bubble plots for pathway analysis.

Metabolites with coefficient of variation (CV) values greater than 30% in QC samples were eliminated from statistical analysis for data quality control. The results of the multivariate statistical analysis are shown in Table 2. The principal component analysis (PCA) model was established to preliminarily understand the overall difference in metabolites and reflect the variation within each group. R^2^X was 0.70 and Q^2^ was 0.41 in the positive ion mode, and R^2^X was 0.79 and Q^2^ was 0.50 in the negative ion mode. Both R^2^X and Q^2^ were greater than 0.4 in the positive and negative ion modes, indicating the reliability of the statistical model. Orthogonal partial least squares discriminant analysis (OPLS-DA) was used for pairwise comparison to observe the separation between groups and aggregation within groups (Figures 9A, B). R^2^X was 0.55 to 0.85, R^2^Y was 0.94 to 1, Q^2^ was 0.62 to 0.96, and all parameters were greater than 0.5, indicating satisfactory stability and prediction ability. The intercepts of Q^2^ on the Y-axis after 200 permutation tests ranged from −0.8 to −0.28, which were less than 0, indicating that OPLS-DA was not overfitting (Figures 3C, D). The variable importance in projection (VIP) values in the OPLS-DA test of the control group (CG), model group (M), and high-dose PGPs group (800) were used to further screen differential metabolites.

**TABLE 2:** The results of multivariate statistical analysis.

Ion mode	Statistical model type	Group	R^2^X	R^2^Y	Q^2^	Int
Positive	PCA		0.70		0.41	
	OPLS-DA	CG vs. M	0.62	0.99	0.82	−0.55
		M vs. 200	0.61	0.98	0.62	−0.47
		M vs. 400	0.65	0.99	0.96	−0.43
		M vs. 800	0.55	0.98	0.71	−0.70
		M vs. Pos	0.61	0.99	0.93	−0.55
Negative	PCA		0.79		0.50	
	OPLS-DA	CG vs. M	0.85	0.94	0.83	−0.80
		M vs. 200	0.80	0.99	0.91	−0.72
		M vs. 400	0.82	1	0.85	−0.31
		M vs. 800	0.76	0.97	0.76	−0.45
		M vs. Pos	0.66	0.99	0.80	−0.28

Int is the intercept of Q^2^ on the Y-axis after 200 permutation tests.

The amplitude of changes in metabolites is shown as volcano plots (Figures 9E, F). According to the criteria of VIP > 1, *P* value < 0.05 of Student’s t test, and fold change (FC) ≥ 1.5 or FC ≤ 0.5, twenty differential metabolites (8 up and 12 down) were screened between CG and M groups. Eighteen differential metabolites (16 up and 2 down) were screened between M and 800 groups. All these differential metabolites were identified by matching with the PubChem (PubChem (nih.gov)), KEGG (KEGG: Kyoto Encyclopedia of Genes and Genomes), and HMDB (Human Metabolome Database (hmdb.ca)) databases. The 10 differential metabolites common to CG vs. M and M vs. 800 are summarized in Table 3. The levels of these common differential metabolites were significantly downregulated in M group compared with CG group, while an upregulation trend was observed after pretreatment with a high dose (800 mg/kg) of PGPs (Figure 9G).

**TABLE 3:** Differential metabolites.

Metabolite name	RT (min)	Adduct type	Mz	Formula	ID	CG vs. M			M vs. 800		
						VIP	P-value	FC	VIP	P-value	FC
D-ribo-Phytosphingosine	1.144	[M+Na]+	340.2817	C18H39NO3	HMDB0004610	1.204951	0.041784	0.002424↓	1.609718	0.005109	7086.62↑
LPC 18:1	14.19	[M+H]+	522.3555	C26H52NO7P	HMDB0002815	1.282675	0.000406	0.335708↓	1.078373	0.026597	2.435479↑
L-Glutamic acid	0.708	[M+H]+	148.0603	C5H9NO4	HMDB0000148	1.241711	0.008268	0.268119↓	1.513421	0.000708	3.227889↑
Cyclopentanone	14.335	[M+NH4]+	102.0913	C5H8O	HMDB0031407	1.49943	2.00E-09	0.315009↓	1.05168	0.02543	1.929284↑
Cyclohexylamine	0.584	[M+H]+	100.1121	C6H13N	HMDB0031404	1.380665	0.003229	0.226286↓	1.212821	0.02728	4.303002↑
Methyl jasmonate	14.306	[M+H]+	225.1487	C13H20O3	HMDB0036583	1.504154	2.72E-08	0.301038↓	1.403339	0.002697	2.47412↑
Serine	2.176	[M+H]+	106.0499	C3H7NO3	HMDB0062263	1.522073	0.000963	0.315705↓	1.723755	1.06E-05	2.204454↑
N-Methylglutamic acid	14.106	[M+H]+	162.0761	C6H11NO4	HMDB0062660	1.559933	3.14E-06	0.234048↓	1.75374	4.96E-07	2.288777↑
Glutamine	0.724	[M+H]+	147.0764	C5H10N2O3	HMDB0000641	1.386633	0.001057	0.174219↓	1.445632	0.001691	3.284965↑
Aspartic acid	2.077	[M+H]+	134.0447	C4H7NO4	HMDB0062186	1.404119	0.004921	0.425436↓	1.137284	0.009719	1.721903↑

The pathway analysis is shown in Figure 9H and Supplementary Table 2. Pathway impact represents the ratio of the number of differential metabolites in a pathway to the total number of metabolites annotated to this pathway; a higher value indicates a greater degree of enrichment. According to the principle of pathway impact > 0.1 and *P* < 0.05, a total of three metabolic pathways were screened, including D-glutamine and D-glutamate metabolism, alanine, aspartate and glutamate metabolism, and arginine biosynthesis.

## 4 Discussion

### 4.1 PGPs exhibit favorable antioxidant ability

Evaluating the antioxidant ability *in vitro* is an effective method to investigate the hepatoprotective activity of PGPs on ALD in the early stage. In our study, the antioxidant ability of PGPs was preliminarily evaluated by measuring the DPPH, OH and O_2_^–^ scavenging rates. Although the effect of PGPs on free radical scavenging was relatively mild compared to the positive control, the dose-dependent free radical scavenging exhibited by PGPs remained excellent. In addition, *Sepia esculenta* ink polysaccharides (SEPs) have been reported to have a favorable antioxidant ability, with IC_50_ values of 20.09 mg/mL for DPPH and 0.33 mg/mL for OH (26). Chestnut polysaccharides (CPs) scavenged free radicals in a dose-dependent manner, and the scavenging rates of DPPH and OH were 88.9% and 41.4% at 10 mg/mL (27). The OH scavenging ability of SEPs and the DPPH scavenging ability of CPs were superior to that of PGPs. PGPs demonstrated a more stabilizing antioxidant ability in DPPH and OH scavenging, with similar IC_50_ values of 6.93 mg/mL and 6.45 mg/mL, respectively. The free radical scavenging ability of bitter gourd polysaccharides have been reported, and the results showed that the highest scavenging rate of DPPH, OH and O_2_^–^ was 39.7%, 36.7%, and 55.0%, respectively, in the concentration range of 0.1 to 3.2 mg/mL (28). Su et al. (29) evaluated the scavenging rate of DPPH, OH and O_2_^–^ by polysaccharides extracted from *Auriculariales* at 0 to 1.2 mg/mL, and the highest scavenging rate of DPPH, OH and O_2_^–^ was 37.8%, 29.7%, 42.4%. In comparison with other polysaccharides, we found that PGPs had superior O_2_^–^ scavenging ability (IC_50_: 0.99 mg/mL), while PGPs had no significant advantage for DPPH and OH scavenging. In summary, antioxidant is an important mechanism for polysaccharide to exert biological activity, so it is necessary to establish ALD cell model to further verify the antioxidant ability of PGPs.

Superoxide dismutase is a major enzymatic antioxidant defense system responsible for scavenging free radicals (30). GSH is an important cellular antioxidant in the body due to its ability to scavenge ROS or as an important cofactor for glutathione S-transferases and peroxidases (31). ROS have the potential to oxidize macromolecules and cause damage to cell membranes, leading to the production of highly reactive peroxides, such as MDA. MDA can cause a cascade of oxidative reactions that inactivate cellular proteins, impair membrane fluidity and elasticity, and ultimately lead to cell death (32). Large amounts of ROS and MDA are released under alcohol intervention, while GSH and SOD react with the free radicals generated by oxidation, resulting in a reduction in their content (Figure 5). Pretreatment with PGPs reduced ROS and MDA production and increased SOD and GSH levels, indicating that the oxidative stress caused by alcohol metabolism was modified. The excellent antioxidant ability conferred the effective preventive potential of PGPs against ALD. It is worth noting that the restorative effect of PGPs on GSH, MDA, SOD, and ROS levels was only dose-dependent at 400–1000 μg/mL. PGPs at 200 μg/mL showed better antioxidant capacity against oxidative stress in the HL7702 cells. Interestingly, Jin et al. reported that *schisandra* polysaccharide increased glucose consumption of buffalo rat liver (BRL) cells in a parabolic fashion at concentrations ranging from 12.5 to 400 μg/mL (33). Maximal glucose consumption was obtained at 100 μg/mL, followed by 200 μg/mL. Glucose consumption is the basis of energy metabolism (34). Natural polysaccharides have also been reported to exert bioactive effects by regulating energy metabolism (35). We hypothesized that with the intervention of 200 μg/mL polysaccharide, cells obtained higher energy metabolism level and thus increased antioxidant ability. However, we have not been able to find more studies to confirm this hypothesis. A deeper research idea should be considered in the future, that is, to explore the mechanism of polysaccharide in alleviating ALD from the perspective of energy metabolism.

### 4.2 Hepatoprotective effects of PGPs in ALD mice are associated with a reduction in inflammatory factors, mitigation of oxidative stress, and regulation of lipid metabolism

ALT, AST, and γ-GGT are metabolic enzymes that function primarily in the cytoplasm of hepatocytes. When the hepatocyte membrane is damaged, ALT, AST and γ-GGT are released into the circulation; therefore, elevated levels in the serum can be used as a marker of liver damage (36). These metabolic enzymes were observed to decrease in the intervention group, indicating that PGPs have the potential to maintain the membrane integrity of hepatocytes damaged by alcohol metabolism. This statement can be confirmed by histopathological observation that the structure of hepatocytes was improved by the intervention of PGPs (Figure 6D).

Liver lipid homeostasis is usually strictly controlled by the metabolic system, and excessive alcohol consumption can easily disturb lipid metabolism and cause rapid reactions such as steatosis (37). Under the interference of alcohol, there was a serious accumulation of lipid droplets in the liver of mice, and TG content increased significantly, *P* < 0.05 (Figures 6E, 7A). PGPs maintained the homeostasis of lipid metabolism in a dose-dependent manner, and the TG content was maintained at a normal level after 800 mg/kg PGPs pretreatment. Polysaccharides can promote fatty acid oxidation to regulate liver lipid metabolism and maintain hepatic stability. In a previous study, Bian et al. (38) used lipidomics to find that *Mori Fructus* polysaccharide had a significant effect on the synthesis and degradation of fatty acids and the metabolism of glycerophospholipids in ALD mice. As one of the phospholipids, glycerophospholipids are the main component of membrane (39). In terms of lipid metabolism, our metabolomic results enriched sphingolipid metabolic pathway and glycerophospholipid metabolic pathway, although *P* > 0.05 (Supplementary Table 2). Our results coincide with the study of *Mori Fructus* polysaccharide, indicating that natural polysaccharide alleviates ALD by regulating lipid metabolism. Furthermore, consistent with *in vitro* experiments, PGPs improved the antioxidant ability of ALD mice, as evidenced by the increase in GSH, SOD, and CAT content and the decrease in MDA content.

In addition, our study showed that the presence of ALD was accompanied by the production of inflammatory factors (Figure 8). Excessive alcohol consumption leads to increased levels of endotoxins, which may be due to altered intestinal flora disrupting the intestinal barrier. Increased intestinal permeability caused by chronic alcohol intake further induces cascading biological effects, including alcoholic liver disease (40). The levels of inflammatory factors (TNF-α, IL-6, LPS) in ALD mice were significantly (*P* < 0.001) decreased by pretreatment with PGPs. Polysaccharides enter the organism and are used as a carbon source by microbial fermentation to maintain homeostasis of the intestinal environment. Wei et al. found that PGPs can be used by intestinal microbiota and can significantly increase the level of short-chain fatty acids during fermentation. The relative abundances of *Bacteroidetes* in PGP groups were increased (2). Astragalus polysaccharides have also been reported to increase the relative abundance of *Bacteroidetes* in ALD mice (41). In addition to *Bacteroidetes*, polysaccharides increased the relative abundance of *Lactobacillus* and *Firmicutes* to alleviate alcohol-induced intestinal barrier damage (42, 43). The improvement of gut microbiota suggests that PGPs can protect ALD mice by reducing the release of endotoxins through maintaining intestinal homeostasis.

Analyzing the alcohol-metabolizing enzymes ADH and ALDH content, we found that there was an increase in the content of ADH and ALDH with the intervention of PGPs, however, it was not significant. We speculated that PGPs alleviated liver damage and thus enhanced the activities of alcohol-metabolizing enzymes, but the protective effect of PGPs on ALD mice was not achieved by increasing the activity of alcohol metabolizing enzymes. In summary, PGPs should be exploited as an excellent anti-ALD bioactive substance, and their hepatoprotective mechanism may be the mitigation of oxidative stress, regulation of lipid metabolism, and reduction of inflammatory factors.

### 4.3 PGPs improve small molecule metabolism in ALD mice

Metabolomics was used to characterize the improvement in the metabolic profile in ALD mice by PGPs pretreatment. In our study, we found that the hepatoprotective effect of PGPs in ALD mice was generally dose-dependent and therefore mainly screened for differential metabolites in the M vs. CG and PGPs-800 vs. M. Pathway analysis was conducted on the ten screened metabolites (Table 3), and three metabolic pathways were screened with a pathway impact > 0.1, as follows: D-glutamine and D-glutamate metabolism, alanine, aspartate and glutamate metabolism, and arginine biosynthesis.

Two metabolites, L-glutamate and L-glutamine, were captured in the D-glutamine and D-glutamate metabolism pathways. L-Glutamine is essential as a metabolic precursor to satisfy the requirements of cell proliferation for peptides and proteins, amino sugars, nucleic acids and nucleotides (44). L-Glutamate is the immediate product of L-glutamine metabolism, which is involved in hepatocyte gluconeogenesis (45). Glutamine and glutamate are involved in a number of biochemical reactions that contribute to tissue repair. In our study, glutamine and glutamate levels were significantly increased (*P* < 0.01) in the PGPs-800 group compared with the M group. We speculate that PGPs regulate glutamine and glutamate metabolism, enhance the functional stability of hepatocytes, and maintain cell membrane integrity, thereby attenuating liver injury. Jiang et al. (43) found that *Echinacea purpurea* polysaccharide could reduce oxidative stress and maintain intestinal permeability in mice with ALD through the D-glutamine and D-glutamate metabolism pathway. In addition, Wang et al. (46) reported that regulation of D-glutamine and D-glutamate metabolism and alanine, aspartate and glutamate metabolism pathways in fatty liver mice was involved in reducing oxidative stress and inflammation, thereby alleviating liver damage. Glutamine exerts potential functions against the harmful effects of oxidative stress (47). D-Glutamine and D-glutamate metabolism pathway is an important metabolic pathway for hepatoprotection. This result verified that the regulation of oxidative stress was a critical mechanism of PGPs against ALD from the metabolic level. Notably, the alanine, aspartate and glutamate metabolism pathways were also enriched in our study. Dai et al. (48) found that alanine, aspartate and glutamate metabolism is an important pathway in the metabolic regulation of *Hedyotis diffusa* in mice with liver injury. Changes in liver metabolism, especially alanine, aspartate and glutamate, will inevitably affect the growth and development of the body (43). In addition, arginine is a multifunctional amino acid, and reduced arginine levels are considered a specific biomarker following liver injury (49). Significant expression of the arginine synthesis pathway indicated that PGPs attenuated liver inflammation in ALD mice. Pretreatment with PGPs alleviates alcohol-induced liver injury by regulating D-glutamine and D-glutamate metabolism, alanine, aspartate and glutamate metabolism, and the arginine biosynthesis pathway to improve liver metabolism.

## 5 Conclusion

Pretreatment with PGPs showed a protective effect against alcohol-induced liver injury. Characterization analysis revealed that PGPs are pyranose polysaccharides mainly composed of arabinose, galactose, xylose, glucuronic acid, mannose, etc. *In vitro* analysis demonstrated that PGPs reduce alcohol-induced oxidative stress in HL7702 cells. In addition, *in vivo* experiments further showed that the hepatoprotective effects of PGPs against alcoholic liver injury were achieved by exerting antioxidant effects, reducing the inflammatory response and regulating lipid metabolism. This study points out that PGPs have a favorable protective effect against ALD, and more in-depth studies such as targeting metabolomics, gut microbiota analysis and transcriptomics deserve to be employed in future studies of PGPs. Overall, PGPs have broad development prospects as highly bioactive and low-cost substance.

## Data availability statement

The original contributions presented in this study are included in this article/Supplementary material, further inquiries can be directed to the corresponding author.

## Ethics statement

Ethical approval was not required for the studies on humans in accordance with the local legislation and institutional requirements because only commercially available established cell lines were used. The animal study was approved by Animal Welfare Ethics Committee of Wuhan Polytechnic University (WPU202209001). The study was conducted in accordance with the local legislation and institutional requirements.

## Author contributions

BZ: Formal Analysis, Investigation, Writing –original draft. PL: Conceptualization, Investigation, Methodology, Writing –review and editing. XY: Writing –review and editing. HC: Writing –review and editing. HZ: Writing –review and editing. QW: Writing –review and editing. YL: Writing –review and editing. MF: Writing –review and editing. YW: Writing –review and editing. ZG: Writing –review and editing.
