# Supplementary material for: Hepatoprotective effects of peach gum polysaccharides against alcoholic liver injury: moderation of oxidative stress and promotion of lipid metabolism
Source: Front Nutr. 2024 Jan 11;10:1325450. doi: 10.3389/fnut.2023.1325450 (PMC10811791; doi:10.3389/fnut.2023.1325450)
Supplement: Supplementary file 1 [file Data_Sheet_1.docx]

***Supplementary Material***

# Supplementary Figures and Tables

## Supplementary Figures


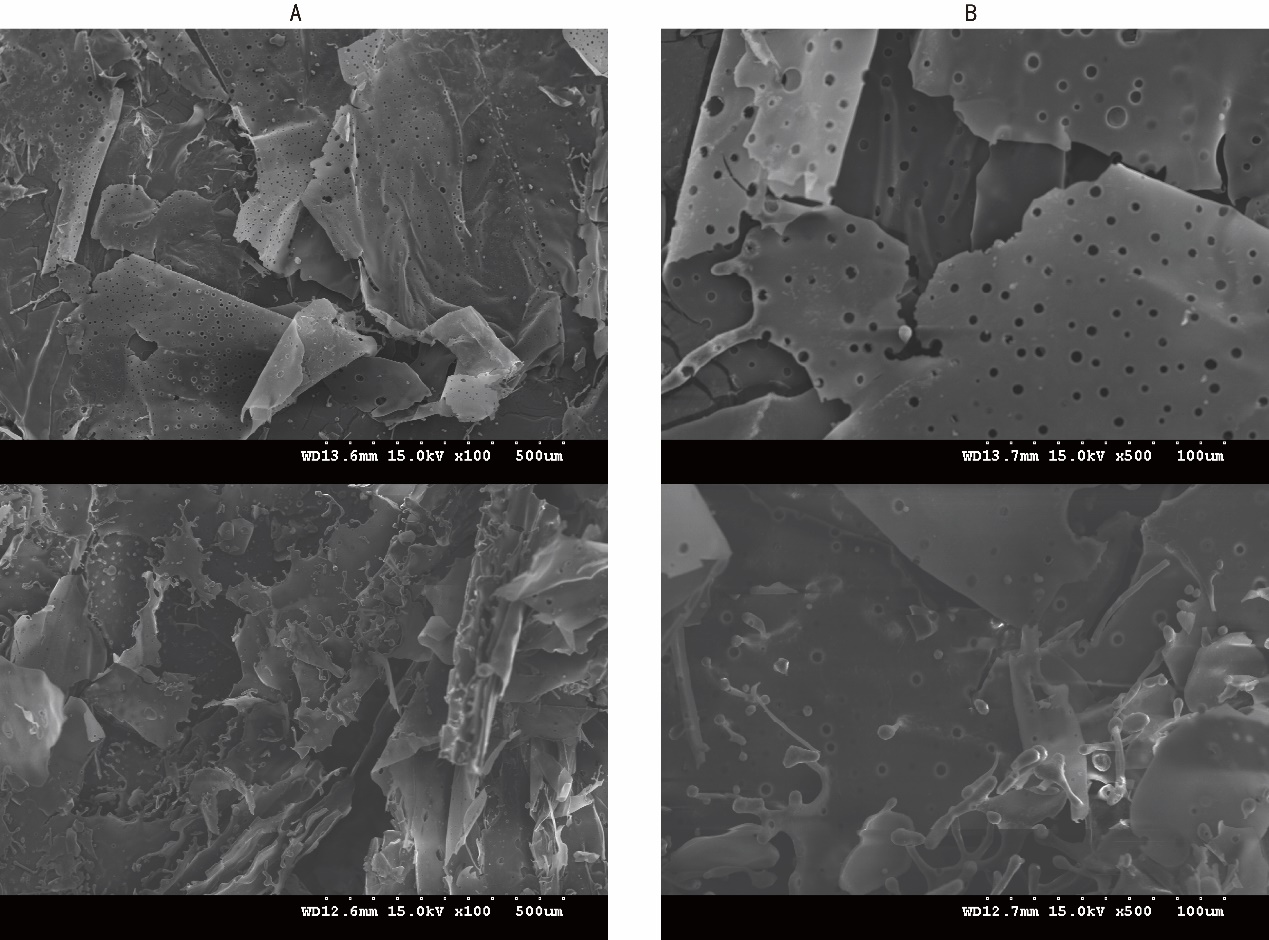


**Figure. S1** Scanning electron microscopy of PGPs. (A) magnification 100×. (B) magnification 500×.


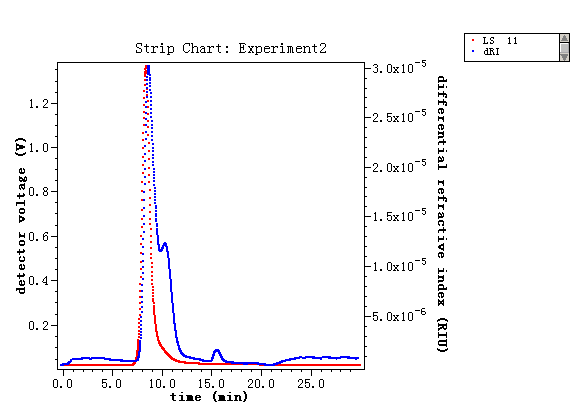


**Figure. S2** HPSEC-RI-MALLS spectrum of PGPs.

## Supplementary Tables

Table S1 Analysis parameter setting

|  | Parameters | |
| --- | --- | --- |
| Data collection | MS1 tolerance | 0.01 Da |
|  | MS2 tolerance | 0.025 Da |
| Peak detection | Minimum peak height | 50000 amplitudes |
|  | Minimum peak width | 5 scans |
|  | Smoothing level | 3 scans |
|  | Mass slice width | 0.1 Da |
| MS2Dec | Sigma window value | 0.5 |
| Identification | Accurate mass tolerance (MS1) | 0.01 Da |
|  | Accurate mass tolerance (MS2) | 0.05 Da |
|  | Identification score cut off | 90% |
|  | Retention time tolerance | 0.5 min |
| Alignment | Retention time tolerance | 0.05 min |
|  | MS1 tolerance | 0.015 Da |
|  | Retention time factor | 0.5 |
|  | MS1factor | 0.5 |
|  | Peak count filter | 14% |
|  | N% detected in at least one group | 100% |

Table S2 The results of pathway analysis

| Pathway name | Match status | P-value | Impact |
| --- | --- | --- | --- |
| Nitrogen metabolism | 2/6 | 7.91×10^-5^ | 0 |
| D-Glutamine and D-glutamate metabolism | 2/6 | 7.91×10^-5^ | 0.5 |
| Arginine biosynthesis | 2/14 | 0.000477 | 0.11675 |
| Alanine, aspartate and glutamate metabolism | 2/28 | 0.001956 | 0.3109 |
| Glyoxylate and dicarboxylate metabolism | 2/32 | 0.002557 | 0 |
| Aminoacyl-tRNA biosynthesis | 2/48 | 0.005731 | 0 |
| Butanoate metabolism | 1/15 | 0.039288 | 0 |
| Histidine metabolism | 1/16 | 0.041865 | 0 |
| Sphingolipid metabolism | 1/21 | 0.054674 | 0.00406 |
| Glutathione metabolism | 1/28 | 0.072391 | 0.01966 |
| Porphyrin and chlorophyll metabolism | 1/30 | 0.077407 | 0 |
| Glycerophospholipid metabolism | 1/36 | 0.092332 | 0.01736 |
| Arginine and proline metabolism | 1/38 | 0.097267 | 0.086 |
| Pyrimidine metabolism | 1/39 | 0.099726 | 0 |
| Purine metabolism | 1/66 | 0.16426 | 0 |
